# Interspecific Gene Flow and Potential Hybrid Breakdown in South Atlantic Sea Turtles

**DOI:** 10.1111/mec.70607

**Published:** 2026-10-24

**Authors:** M. D. Oliveira, R. J. Pereira, L. S. Arantes, C. J. Mazzoni, F. R. Santos

**Affiliations:** ^1^ Laboratório de Biodiversidade e Evolução Molecular (LBEM) Instituto de Ciências Biológicas, Universidade Federal de Minas Gerais Belo Horizonte Minas Gerais Brazil; ^2^ State Museum of Natural History Stuttgart Stuttgart Germany; ^3^ University of Hohenheim Stuttgart Germany; ^4^ Center of Excellence for Biodiversity and Integrative Taxonomy (KomBioTa) Stuttgart Germany; ^5^ Leibniz Institute for Zoo and Wildlife Research (IZW) Berlin Germany; ^6^ Berlin Center for Genomics in Biodiversity Research (BeGenDiv) Berlin Germany; ^7^ IN2PAST.BR – National Institute of Science and Technology in Natural and Cultural Heritage Universidade Federal de Minas Gerais Belo Horizonte Minas Gerais Brazil

**Keywords:** cheloniidae, hybridisation, population genomics, reproductive barriers

## Abstract

Interspecific hybridisation can play an important evolutionary role, generating new taxa or outbreeding depression, which can also lead to behavioural reproductive isolation through reinforcement. Still, there are few studies investigating how divergence time relates to hybridisation rates and its impact on introgression. Sea turtles are known to hybridise, despite their long divergence times and behavioural, ecological and morphological differences. Several genetic studies have attempted to determine the extent of hybridisation between four of the seven extant sea turtle species ( *Caretta caretta* , *Eretmochelys imbricata* , *Lepidochelys olivacea* and *Chelonia mydas* ) in the South Atlantic coast of Brazil, an area known to include nesting and feeding sites for all these species. In this work, we combined new and published genomic data of all four sea turtle species and their hybrids in a detailed population genomics study. Although hybridisation does occur in Brazilian nesting sites between three of the four species, it appears to be limited to first‐generation hybrids and few backcrosses, which points to some postzygotic barriers between species and hybrid breakdown. Moreover, our evidence suggests a moderately high level of relatedness among hybrids, indicating a reduced number of independent hybridisation events. Despite this fact, we found evidence of backcrosses involving different species, and an F2 progeny between *Ca. caretta* x *E. imbricata* F1 hybrids, which suggests hybrid male fertility. Limited interspecific gene flow appears to be part of the long history of divergence of sea turtles, as low levels of deep time introgression were inferred. In summary, this work contributes important evidence regarding the rate and success of hybridisation at large scales of divergence, and how this seemingly equates to low rates of introgression, pointing to temporal limits to gene flow.

## 1 Introduction

Hybridisation has recently been recognised as a relatively common phenomenon that arises when two separated populations or species interbreed (Peñalba et al. 2024), being now estimated to occur in as much as 10% of vertebrate species (Moran et al. 2021; Seehausen 2004). In many cases, hybrid offspring are expected to be the carriers of deleterious combinations of alleles that would render them unfit for survival or sterile—with some being called “evolutionary dead‐ends” (Adavoudi and Pilot 2021; Bierne et al. 2002; Pala and Coelho 2005; Zhang et al. 2022). Successful rounds of hybridisation with backcrossing could result in the introduction of genetic material from one species into the genome of another, a phenomenon known as introgression (Aguillon et al. 2022). Not all hybridisation results in introgression, as the latter depends on the fertility of hybrids, the compatibility between hybrids and parental species and their overall reproductive success. For example, hybridisation may occur even in a scenario of strong reproductive barriers to gene flow, being restricted only to the first generation of hybrids. However, it is still unclear whether hybridisation between deeply divergent lineages is accompanied by significant introgression that can shift the evolutionary trajectories of species.

Successful hybridisation between two taxa is expected to be negatively correlated with their time of divergence, although the timing at which gene flow ceases completely between different species can vary, sometimes taking several million years, such as in the case of gars and sturgeons (Brownstein et al. 2024). Furthermore, reproductive barriers seem to arise later in animal species when compared to plants, which could indicate that hybridization in animals can also happen between deeply divergent species (Monnet et al. 2025). Still, not much is known about the success of hybridisation at large divergence timescales, and whether reinforcement is a common phenomenon that arises when hybrid fitness is reduced—questions that can be investigated by studying deeply diverged taxa that hybridise in nature.

The common ancestors of living turtles (order Testudines) are estimated to have appeared around 220 million years ago (Mya), during the Late Triassic (Shaffer et al. 2017). Only seven species of sea turtles are still extant: the loggerhead *Caretta caretta* (Linnaeus, 1758), the hawksbill *Eretmochelys imbricata* (Linnaeus, 1766), the green turtle *Chelonia mydas* (Linnaeus, 1758), the olive and Kemp's ridleys *Lepidochelys olivacea* (Eschscholtz, 1829) and *L. kempii* (Garman, 1880), the flatback turtle *Natator depressus* (Garman, 1880) and the leatherback *Dermochelys coriacea* (Vandelli, 1761); all of those species are members of the superfamily Chelonioidea, and their divergence times vary between approximately 10 My and 80 My (Arantes et al. 2025; Vilaça et al. 2021). These animals are long‐lived, possess lengthy generation times and a slow evolutionary rate, which may partially explain their highly conserved genomes with a well‐maintained synteny (Arantes et al. 2025). Ecologically, sea turtles are well known for their long‐ranged migratory behaviour, and while juveniles are pelagic, the females are highly philopatric—which may also apply to male adults—returning to the same coastal area of birth during the nesting seasons (Bowen and Karl 2007; FitzSimmons et al. 1997; Lohmann et al. 1999; Papi et al. 2000).

The extant species of sea turtles are considered either vulnerable, endangered, or data‐deficient by the IUCN (IUCN 2008a, 2008b, 2013, 2015, 2019, 2021) and conservation efforts of these animals are commonplace around the world. On the eastern Brazilian coast, where many beaches and few islands are well‐known nesting areas for different species of sea turtles, conservation management has been employed since the 1980s (Marcovaldi and Marcovaldi 1999). Previous works were able to genetically characterise the Brazilian sea turtle populations and identified an extensive record of hybridisation between four of those species—namely *Ca. caretta*, *Ch. mydas*, *E. imbricata* and *L. olivacea* (Arantes, Vilaça, et al. 2020; Lara‐Ruiz et al. 2006; Reis et al. 2010). Typical interspecific hybrids are usually first generation (F1) sea turtles, although certain hybrid zones also present few backcrossed and second generation (F2) individuals (Arantes, Ferreira, et al. 2020; Morais et al. 2026; Vilaça et al. 2023). It is still unclear, however, which taxa are involved in hybridisation and whether this leads to introgression.

Hybridisation in these four species of sea turtles has been documented and studied genetically, but previous studies lacked the power to infer some specific hybrid crosses and the long‐term history of hybridisation. Furthermore, it is yet unknown whether the strength of interspecific gene flow is correlated negatively with the divergence time between sea turtle species, whether there is a clear directionality in hybrid crossings, and whether hybridisation equates to gene flow at these evolutionary timescales. These gaps could be fulfilled by the integration of a comprehensive genomic dataset, together with the use of high‐quality reference genomes, which should allow for more precise comparative analyses at the genomic level and deep inferences regarding the timing and strength of hybridisation and gene flow among sea turtles.

In this work, we collected new genomic sequences of reduced‐representation libraries and previously published data for a total of 167 samples of sea turtles across four species of Cheloniidae: *Ca. caretta*, *Ch. mydas*, *E. imbricata* and *L. olivacea* . Our goal is to integrate this large dataset, together with new high‐quality reference genomes for all species, to ask how the rates and success of interspecific hybridization and introgression are affected by long divergence times in vertebrates.

## 2 Methods

### 2.1 Genetic Samples

We have generated new 3RAD sequences (see protocol below) for 15 *Ca. caretta*, one *Ch. mydas*, seven *E. imbricata* , 23 hybrids previously confirmed through molecular identification (Arantes, Ferreira, et al. 2020; Arantes, Vilaça, et al. 2020; Vilaça et al. 2023) and 46 individuals with no previous molecular identification from six Brazilian states, from the Elevação do Rio Grande (ERG) feeding area in the Southeast Atlantic and from Guadeloupe Island (Table 1). These new samples were selected to complement previous RADseq data generated by our group (Arantes, Ferreira, et al. 2020; Vilaça et al. 2023), comprising localities of Martinique Island (Caribbean), French Guiana and Brazilian sites of Espírito Santo and the Abrolhos Archipelago (Bahia), representing a total sample of 167 individuals (Figure 1). All new DNA sequences were obtained from previously deposited tissue samples in the Centro de Coleções Taxonômicas (CCT) of the Universidade Federal de Minas Gerais (UFMG). Access to genetic resources is registered at Sisgen/Brazil under registration number A03A2C2, according to the Nagoya Protocol.

**TABLE 1:** Number of 3RAD samples generated in this study per species and sampling site.

Site	Cc	Cm	Ei	Hybrid	Unknown
Bahia	9	0	3	20	9
Sergipe	2	0	0	2	20
Pernambuco	0	0	2	0	1
Rio Grande do Norte	0	0	2	0	4
Elevação do Rio Grande	3	0	0	0	9
Guadeloupe	0	1	0	0	0
Rio de Janeiro	1	0	0	0	3
São Paulo	0	0	0	1	0
Total	15	1	7	23	46

Abbreviations: Cc: Caretta caretta; Cm: Chelonia mydas; Ei: Eretmochelys imbricata.

**FIGURE 1:**
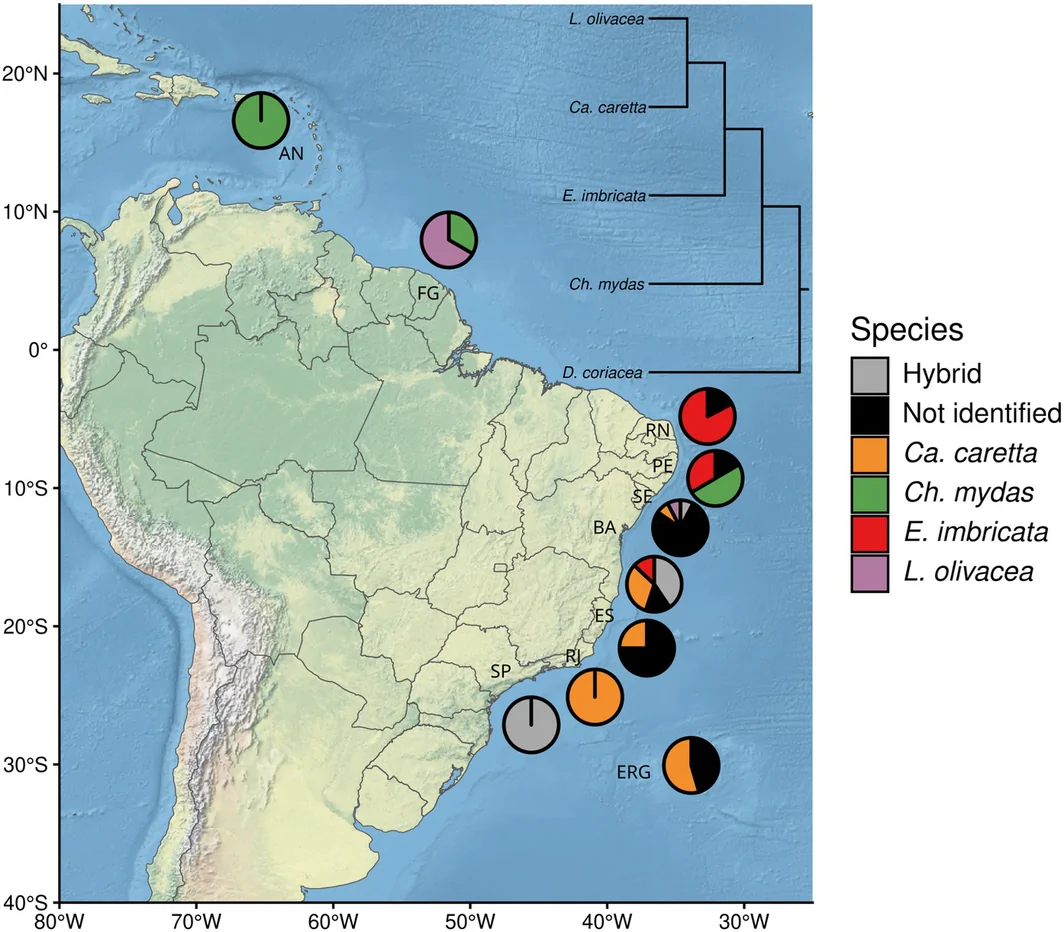
Map depicting approximate sampling locations for all samples. Pie chart sections are coloured according to previous molecular identification of samples (Arantes, Ferreira, et al. 2020; Arantes, Vilaça, et al. 2020; Vilaça et al. 2023). Localities are depicted as FG (French Guiana), AN (Antillean Islands of Guadeloupe and Martinique) and the Brazilian sites of BA (Bahia), SE (Sergipe), PE (Pernambuco), RN (Rio Grande do Norte), ERG (Elevação do Rio Grande), ES (Espírito Santo), RJ (Rio de Janeiro) and SP (São Paulo). The inset dendrogram shows the relationship between the four studied species and the outgroup *Dermochelys coriacea* .

### 2.2 3RAD Sequencing and Preprocessing

The library construction and preprocessing were performed as described by Arantes, Ferreira, et al. (2020). Briefly, enzymes *Mse*I, *Eco*RI and *Cvi*QI were used to digest the genome and DNA fragments between 350 and 450 bp, which were selected to be sequenced. Sequences were trimmed to keep only high‐quality data (PHRED score > 30, minimum length 130 bp, undigested and chimaera removal, PCR duplicates removal).

We incorporated ddRAD sequences for 75 previously published samples of sea turtles (Vilaça et al. 2023). These were produced with the same cut enzymes (i.e., *Mse*l and *Eco*RI) as our 3RAD samples, giving us enough confidence on the feasibility of combining both datasets. We confirmed it by evaluating the distribution of insert sizes of the two methods and by computing the overlap of positions covered by at least five reads for the two methods.

The final dataset comprises pre‐processed sequencing reads for 167 samples (Table S1) with previous molecular identification of *E. imbricata* (*N* = 30), *Ca. caretta* (*N* = 45), *L. olivacea* (*N* = 6), *Ch. mydas* (*N* = 7), hybrid (*N* = 31) or lacking molecular identification (*N* = 48).

### 2.3 Reference Mapping and Genotype Calling

We mapped all sequencing reads to the primary assembly of the *D. coriacea* (RefSeq accession GCF_009764565.3) reference genome (Bentley et al. 2023), to avoid potential biases arising from mapping to an ingroup species (Cheloniidae), using the algorithm implemented in *bwa‐mem2* v2.2.1 (Vasimuddin et al. 2019) with default parameters. In addition, we tested mapping the reads to the primary assemblies of all other six reference genomes to assess potential biases arising from mapping to a reference species that diverged as much as 80 Mya from the ingroup. To look for the potential mapping bias, we evaluated the weighted mean mapping depth across all seven reference genomes for all samples as reported by the *gstacks* module of *stacks* v2.68 (Catchen et al. 2013), looking for obvious patterns in the coverage distribution that would indicate biases during mapping. For assessing reference biases and for the final mapping, we used *samtools* v1.20 (Li et al. 2009) to keep only reads with a mapping quality score > = 30 (*−q 30*), only properly paired reads (*−f 0 × 2*) and no secondary/supplementary hits (*−F 0 × 900*).

We then used the *bcftools* v1.20 *mpileup*/*call* pipeline (Danecek et al. 2021) for genotype calling and *VCFtools* v0.1.17 (Danecek et al. 2011) for filtering the calls. Briefly, we only kept calls that were not located in known repeat regions of the reference genome, in the primary scaffolds representing the 28 sea turtle chromosomes, that had a minimum call quality score of 20, a calling depth between 10 and 100, with a maximum of 20% missing data, and only kept SNPs with at most two alleles whilst removing indels. In total, we generated two distinct datasets suited for different analyses: (1) the *All* dataset, containing all variant and invariant sites; and (2) the *LD_pruned* dataset, containing biallelic SNPs that were filtered for linkage disequilibrium using *plink* v2.0.0 (Chang et al. 2015) with the function *–indep‐pairwise* within a window of 50 SNPs, with a step size of 10 SNPs and a correlation coefficient of 0.1.

Additionally, since we wanted to perform also long‐term introgression analyses, which require an outgroup to be present, we used ddRAD‐seq data (Driller et al. 2021) from two samples of *D. coriacea* available on NCBI (BioProject PRJNA862341). This data was produced using the same cut enzymes as the rest of our data, making integration seamless. Reads were quality‐checked using *FastQC* v0.12.1 (Andrews 2010) and sequences were trimmed using *fastp* v0.23.4 (Chen et al. 2018). We then mapped the ddRAD reads to the *D. coriacea* reference genome and proceeded with variant calling as described above.

### 2.4 Admixture Analysis

To evaluate the degree of admixture between species, we employed three distinct strategies based on the *LD_pruned* dataset. First, we used a PCA analysis as a non‐parametric approach implemented in *plink* with 20 principal components. Under this analysis, we expect the first few principal components to reflect the phylogenetic divergence between species, separating the most divergent ones, with hybrids appearing in between the hybridising taxa (parental species).

Secondly, we used *ADMIXTURE* v1.3.0 (Alexander et al. 2009) to perform a maximum‐likelihood based ancestry estimation for all samples. *ADMIXTURE* was used to calculate the ancestry proportions for an individual assuming *K* putative ancestral clusters, where *K* is supplied by the user. We tested *K* values from one to ten (representing all species and sampling localities), and for each value of *K*, we ran 20 independent replicates each using a cross‐validation (CV) procedure as implemented in *ADMIXTURE* to evaluate its fit to the data. We then selected the run with the lowest CV error per value of *K* as the representative run for that *K* and chose the best value of *K* based on the overall lowest CV error.

Third, to further evaluate the extent of admixture and assign generations to the putative hybrids, we ran a hybrid index analysis using *triangulaR* v0.0.1 (Wiens et al. 2025). This software performs an estimation of hybrid index and interclasses heterozygosity based on called genotypes, which can then be visualised as a triangle plot where the placement of each sample is an indication if it is a first‐generation hybrid (F1), a first generational backcross (BC) with either parental species, a F2 hybrid (crossing between F1 hybrids), or a later generation hybrid. We generated sample subsets of the dataset containing pairs of parental species and their hybrids as assessed by the *ADMIXTURE* analysis and excluding all sites with missing data for the sample subset. Based on the *ADMIXTURE* results for the best value of *K*, we defined a sample as a member of a given species if it had a single ancestral component with a proportion > = 95%, or as a hybrid of a given pair of parental species if the sum of the proportions of the admixture components of both species was > = 95% while each individual contribution was > = 5%. For each possible pairing of *Ca. caretta*, *E. imbricata* and *L. olivacea* , we used *triangulaR* to select SNPs with fixed allelic differences (allelic difference threshold = 1), to estimate hybrid indices and heterozygosities and to generate the triangle plots. Since no hybrids involving *Ch. mydas* or the *Ca. caretta* from ERG were identified in our dataset (see Results), we excluded them from this analysis.

Lastly, to quantify and compare the levels of genomic diversity within species, we calculated summary statistics for each species using the *All* dataset and the R package *GenoPop* v1.0.0 (Gurke 2024). First, we calculated kinship values for all samples using *plink* and removed all but the sample with the highest weighted mean depth among related samples, using as a threshold a kinship coefficient ≥ 0.25. We then classified samples as described above, based on the *ADMIXTURE* results. Since *Ca. caretta* was divided into two populations when *K* = 5 (see Results), the diversity statistics were also calculated for each population separately. Using *GenoPop*, we calculated *π*, the observed heterozygosity, Watterson's θ and Tajima's D. Additionally, to elaborate on the divergence between species and the two identified *Ca. caretta* populations (see Results), we calculated F_ST_ and D_XY_ values using *GenoPop*.

### 2.5 Demographic Models

To evaluate the most likely scenario regarding the mode of speciation, the timing and rate of gene flow between species, we ran a demographic analysis using the diffusion approximation algorithm implemented in *dadi* v2.4.3 (Gutenkunst et al. 2009), which models demographic processes of a pair of populations based on the joint site frequency spectrum (jSFS).

Based on the *All* dataset, we computed the best projections for each species pair using *easySFS* (https://github.com/isaacovercast/easySFS) and used those values to generate the folded jSFS using *dadi‐cli* v0.9.14 (Huang et al. 2023). For all pairs involving *Ca. caretta*, we excluded the individuals identified as being from the ERG feeding area, since no admixture involving those individuals was identified and because population structure may bias demographic inferences. Using *dadi‐cli*, we compared five demographic models of varying complexity: (i) split with no gene flow (“No Migration”, with parameters *Nu1* and *Nu2* as the effective population size of each species, and *T* as the time of divergence); (ii) split with continuous, symmetrical gene flow (“Symmetric Migration”, with an additional parameter *m*, the migration rate); (iii) split with symmetrical gene flow during secondary contact (“Secondary Contact”, with additional parameters *m* for the migration rate and *T2* for the time of secondary contact); (iv) split with asymmetrical gene flow (“Asymmetric Migration”, with parameters *m12* and *m21* as the respective migration rates); and (v) split with asymmetrical gene flow on secondary contact (“Secondary Contact Asymmetric Migration”). We estimated model parameters in a total of 100 independent runs for each model, using 25% of those runs for global optimisation and the rest for local optimisation to ensure convergence. We set the lower boundaries for the parameters *Nu1*, *Nu2*, *m* (or *m12* and *m21* for the asymmetrical gene flow models), *T1* and *T2* as 1 × 10^−3^, 1 × 10^−3^, 0, 0 and 0 and the upper boundaries as 2, 2, 5, 10 and 10, respectively. Run results were manually inspected for convergence after running three independent chains using the *BestFits* function of *dadi‐cli*.

To account for the effects of linkage, we generated 100 non‐parametric bootstrapped jSFS for each population pair using *dadi‐cli* and employed a Godambe Information Matrix‐corrected likelihood ratio test (LRT) to perform model selection (Coffman et al. 2016). Models were compared as follows: symmetric migration × no migration; asymmetric migration × symmetric migration; secondary contact × symmetric migration; secondary contact asymmetric migration × asymmetric migration. Using the same bootstrapped jSFS, we also estimated the parameter uncertainty and calculated confidence intervals for the parameters of the best fit model for each comparison. We converted parameter estimates to real units using a mutation rate of 7.9 × 10^−9^ substitutions/site/generation, calculated for reptiles (Green et al. 2014).

### 2.6 Introgression

To look for additional evidence of long‐term interspecific gene flow, we used an *F*_*branch*_ statistics test using *Dsuite* v0.5 (Malinsky et al. 2021). For this analysis we utilised a dataset consisting of 192,765 biallelic SNPs among all pure samples and the outgroup *D. coriacea* . We ran *Dsuite Dtrios* with default parameters to calculate the proportion of ABBA‐BABA sites and the *f*_*4*_ ratio and then used the *Dsuite Fbranch* command with a significance value of 0.05 to calculate *F*_*branch*_ statistics.

## 3 Results

### 3.1 Reference Mapping and Data Filtering

Our insert length analysis showed an overlap of fragment sizes between RAD libraries, which indicates similar—although not equal—size selection strategies (Figure S1). Additionally, we quantified the overlap of positions where > = 5 reads mapped to the *D. coriacea* reference genome and found that a total of 28,253,981 unique sites (36% of all evaluated sites) overlapped between libraries, a number considered sufficient to ensure variant calling that would not be negatively impacted by combining datasets (Figure S2).

All 167 samples were mapped to the *D. coriacea* reference genome with a mean mapping depth > = 5 (Table S2). Mapping depth did not seem to vary significantly between all possible reference genomes (Figure S3). We then called genotypes for all those samples jointly, which after filtering resulted in 1,065,048 sites in the *All* dataset and 193,233 biallelic SNPs, of which 20,675 are present in the *LD_pruned* dataset.

### 3.2 Admixture Analysis

The PCA analysis was able to separate all four species into distinct clusters (Figure 2A), with putative hybrids appearing as intermediates between pairs of species. The PC1 explained 28.5% of the total variance while PC2 explained 19.9%. When excluding *Ch. mydas* from the dataset, a triangle‐shaped pattern is observed between the remaining species and their hybrids (Figure 2B).

**FIGURE 2:**
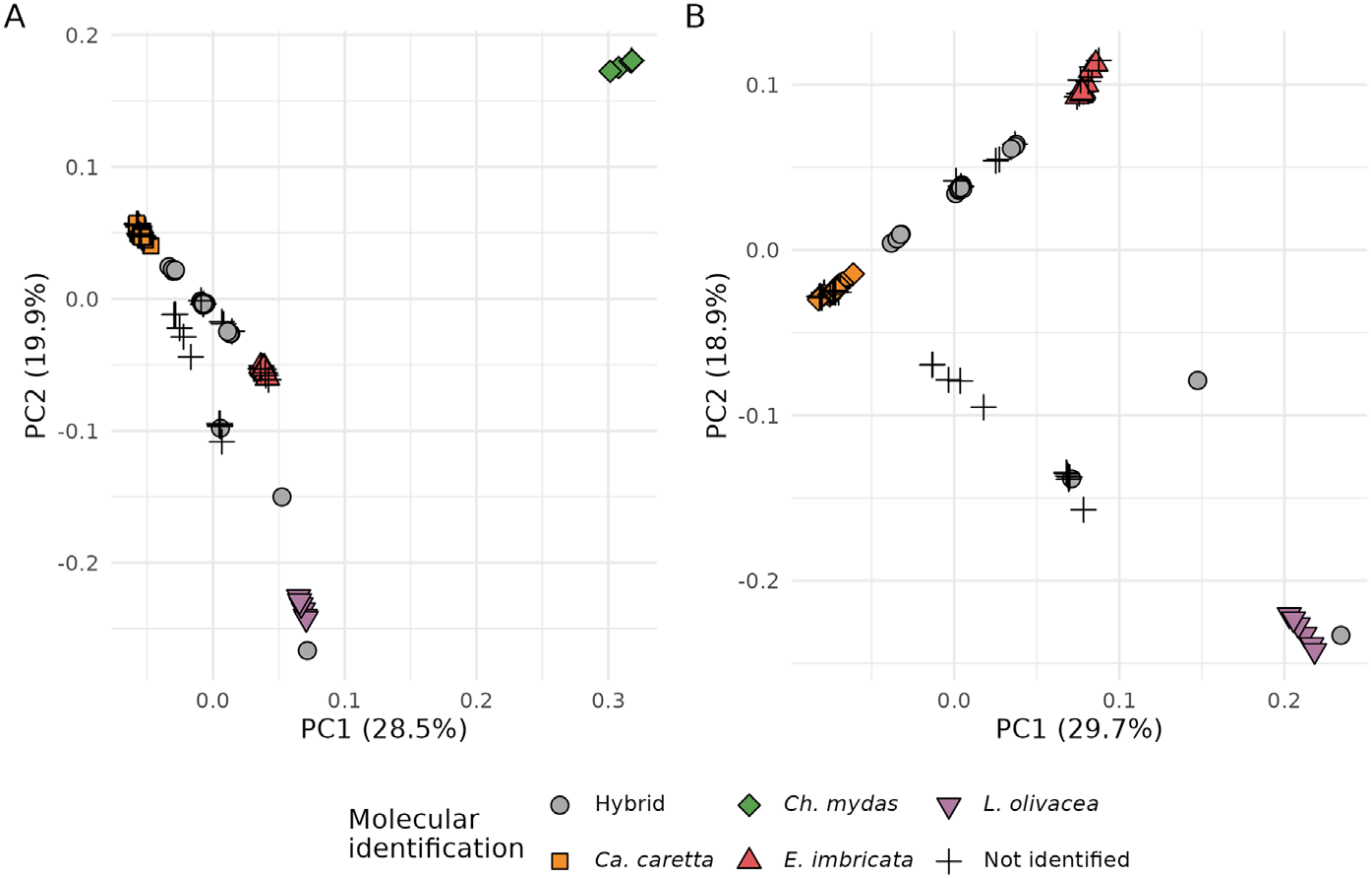
PCA for the first two principal components, (A) including *Ch. mydas* and (B) excluding it. Samples are coloured according to previous molecular identification (Arantes, Ferreira, et al. 2020; Arantes, Vilaça, et al. 2020; Vilaça et al. 2023).

For the *ADMIXTURE* analysis, using a cross‐validation procedure, we found that *K* = 5 is the best supported value of *K* (Figure S4). All four species are well separated when *K* = 4 (Figure 3), which is reflective of the structure observed in the PCA. Furthermore, at *K* = 5, *Ca. caretta* is separated into two distinct ancestry clusters; this pattern is also observed when plotting PC3 and PC4 (Figure S5). We are also able to identify hybrids between all combinations of *Ca. caretta*, *E. imbricata* and *L. olivacea* within the samples previously confirmed as hybrids and within the non‐identified samples. In total, we identified 49 individuals as hybrids. No hybrids involving *Ch. mydas* or the *Ca. caretta* from ERG were identified among our samples (Figure 3; Table S3).

**FIGURE 3:**
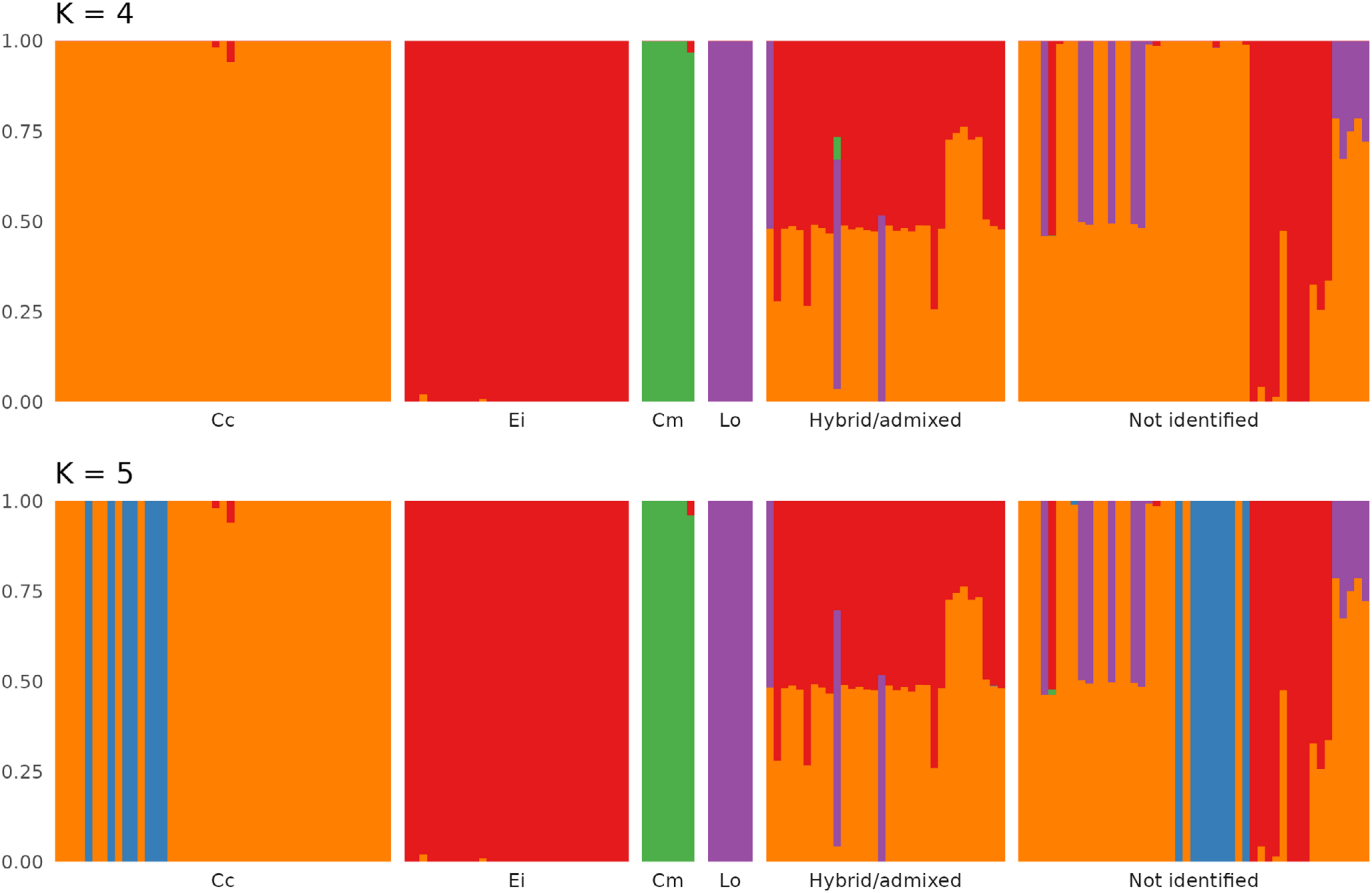
ADMIXTURE plot showing the ancestry proportions for each sample for *K* = 4 and *K* = 5. Labels are assigned to samples based on prior molecular identification (Arantes, Ferreira, et al. 2020; Arantes, Vilaça, et al. 2020; Vilaça et al. 2023).

The triangle plots (Figure 4) show that all species pairs involving *Ca. caretta* produce F1 hybrids and first‐generation backcrosses. In the pairing *Ca. caretta* and *E. imbricata* , based on 1312 sites, we also find evidence of a putative later‐generation backcross, an F2 hybrid and a potential backcross of an F2 hybrid with *E. imbricata* (Table S4). Sample T250BAABPCC appears to be an F2 hybrid, while T196BAPDFEI could be, arguably, the result of a backcross of an F2 hybrid individual with *E. imbricata* . Sample T101BAABNA was defined as introgressed between *Ca. caretta* and *E. imbricata* and appears to be a late generation backcross in the triangle plot; however, other samples from the same nest as this individual (i.e., siblings) are identified as being pure *Ca. caretta*. The interbreeding of *E. imbricata* and *L. olivacea* , based on 4926 sites, only produces F1 hybrids (Table S5). Lastly, the pairing between *Ca. caretta* and *L. olivacea* , based on 4113 sites, produces mostly F1 hybrids, with a few first generation of backcrosses, all coming from the same nest (i.e., all siblings; Table S6). All individuals identified as backcrosses in all pairings are hatchlings (Table S1).

**FIGURE 4:**
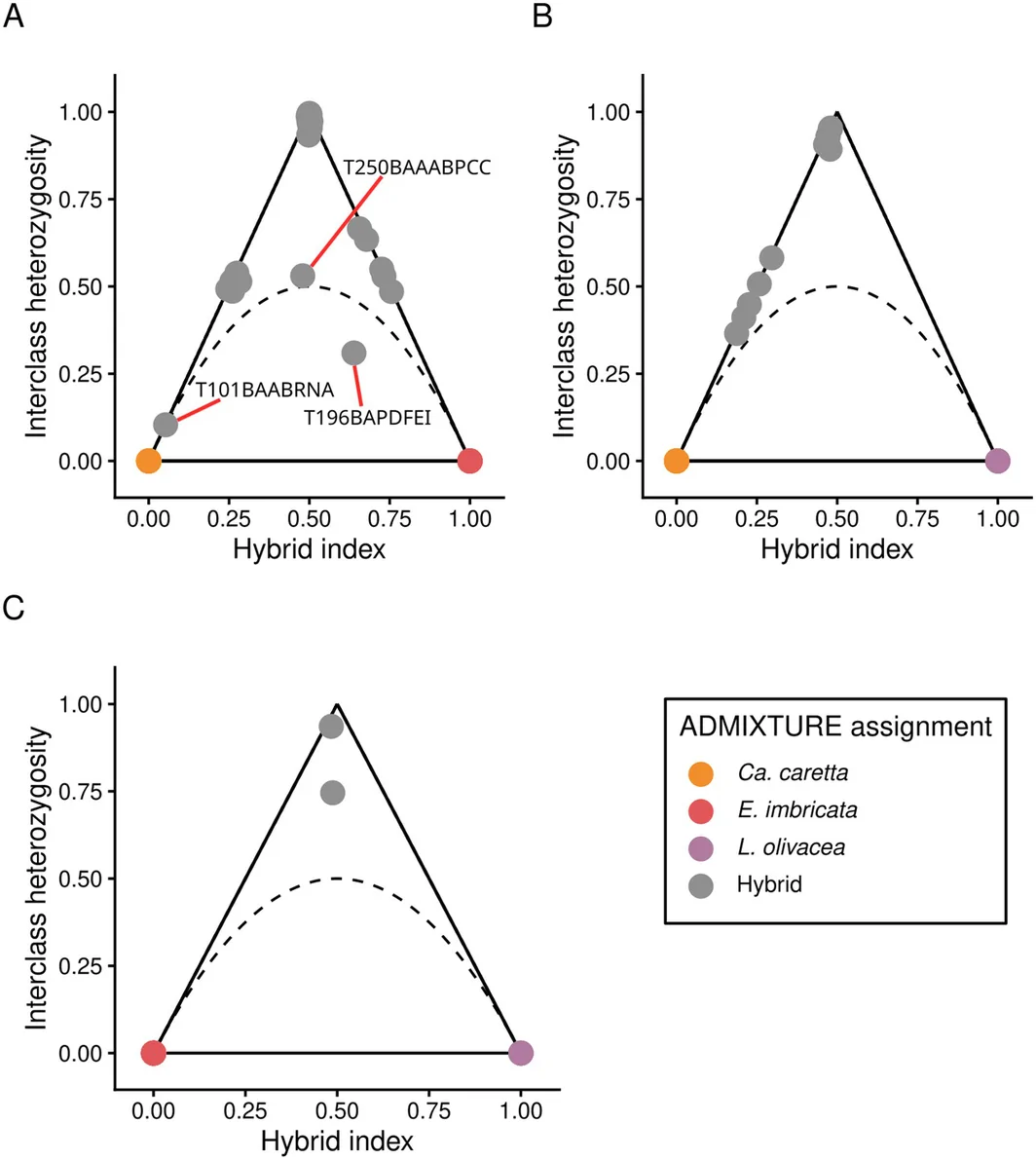
Triangle plots showing the generations of hybrid crossings between *Ca. caretta* and *E. imbricata* (A), *Ca. caretta* and *L. olivacea* (B), and *E. imbricata* and *L. olivacea* (C).

Most related samples are classified as “hybrid”; among those, 35 samples are highly related (kinship > = 0.25) to at least one other sample (Table S7). The only related samples among individuals classified as “pure” were a triplet of *L. olivacea* samples (Table S7). To avoid downward biassing our diversity estimates, we only kept the “best sample” (based on the weighted mean mapping depth) among that triplet. Between all evaluated summary statistics, we observe that *Ch. mydas* shows the highest genetic diversity, followed by *E. imbricata* , *L. olivacea* and the two populations of *Ca. caretta* (Table 2). All species have a negative Tajima's D, which could be an indication of population expansion (Table 2). Divergence is comparably high between species and much lower among *Ca. caretta* populations (Table S8). Interestingly, F_ST_ between *Ca. caretta* and *E. imbricata* was lower than between the sister species *Ca. caretta* and *L. olivacea* , although D_XY_ values were more in line with phylogenetic divergence.

**TABLE 2:** Summary statistics for all species and the two populations of *Ca. caretta*.

Population	π	Heterozygosity	θ	Tajima's D
Lo	0.0025	0.0032	0.0027	−0.3429
Cc BR	0.0025	0.0025	0.0030	−0.5173
Cc ERG	0.0023	0.0021	0.0027	−0.5491
Cm	0.0116	0.0134	0.0127	−0.4004
Ei	0.0046	0.0045	0.0056	−0.6211

Abbreviations: Lo: L. olivacea; Cc BR: Ca. caretta Brazil; Cc ERG: Ca. caretta Elevação do Rio Grande; Cm: Ch. mydas; Ei: E. imbricata.

### 3.3 Demographic Models

For all population pairs, model likelihood is highest for the models of asymmetric gene flow at secondary contact. When comparing models with a penalised LRT, a model of continuous symmetrical gene flow is significantly better than a model of no gene flow, while a model of continuous asymmetrical gene flow is consistently better than simpler models, with the exception of the *Ch. mydas* x *L. olivacea* pair (Table S9; Figure S6), where the small sample sizes could be limiting model estimation. To provide adequate comparisons, we therefore estimated the real parameters for both models (i.e., symmetrical and asymmetrical continuous gene flow) and compared species pairs within each model separately.

Within the asymmetrical gene flow model, divergence times are largely concordant with the phylogenomic estimates for the species (Arantes et al. 2025; Vilaça et al. 2021). The same is not true for the symmetrical migration model, where divergence times between *Ca. caretta* and *L. olivacea* seem to be overestimated, and divergence between *Ca. caretta* and *E. imbricata* , *Ch. mydas* and *L. olivacea* , and *E. imbricata* and *L. olivacea* seem to be underestimated. For the asymmetrical migration model, the migration rates were the highest in the sister species *Ca. caretta* and *L. olivacea* , with a strong directionality going from *Ca. caretta* to *L. olivacea* , which contrasts with the directionality observed in our triangle plots (Figure 4). Importantly, all effective migration rates (N * m) were much lower than 1, suggesting that gene flow was very restricted among sea turtle species. Effective population sizes were congruent with our diversity estimates: higher in *Ch. mydas* and *E. imbricata* , and lower in *Ca. caretta* and *L. olivacea* (Figures 5 and Figure S7; Table S10).

**FIGURE 5:**
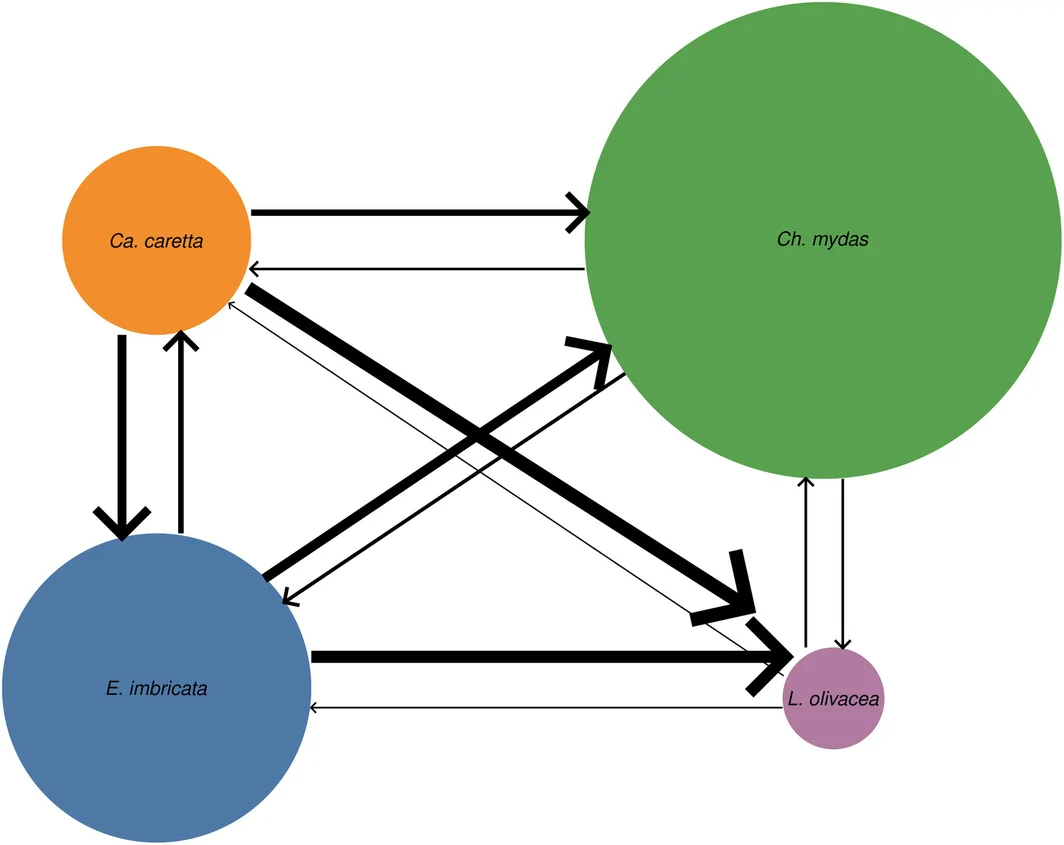
Schematic representation of the demographic models. Circles represent each species, with size proportional to the mean population size across all three models containing each species. Arrows represent directional migration, with width proportional to the migration rate.

### 3.4 Introgression

The *F*_*branch*_ test estimated a proportion of ~1.6% of introgression between *Ca. caretta* and *E. imbricata* and approximately 0.2% introgression between *E. imbricata* and *Ch. mydas* (Figure 6). No significant amount of introgression was observed among any other species pairs.

**FIGURE 6:**
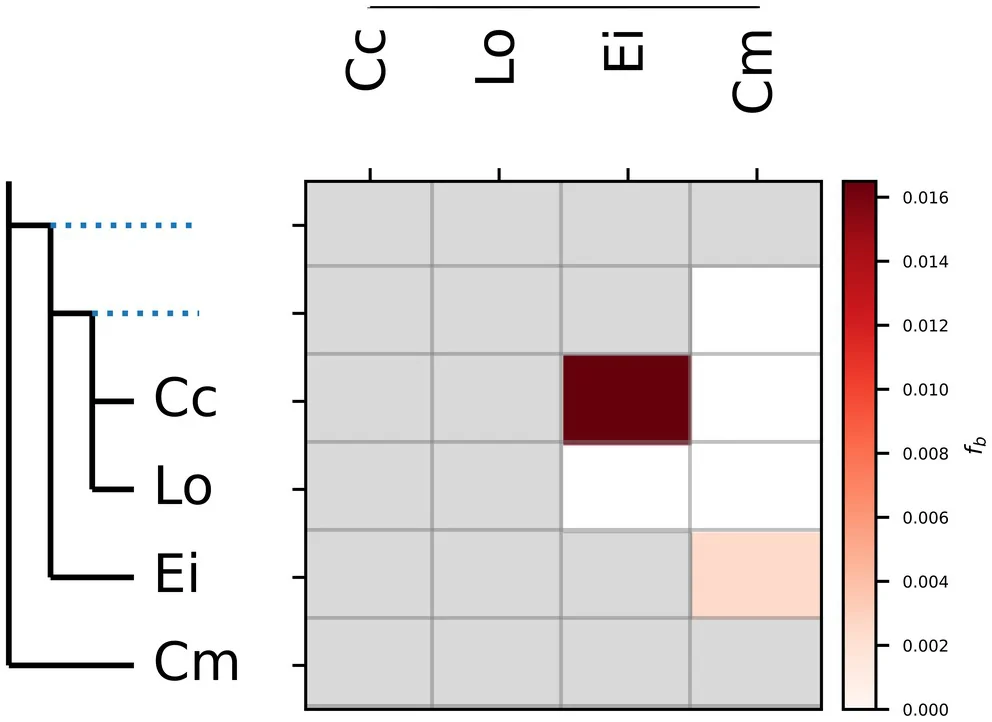
F_branch_ values for all tested comparisons. Darker colours signify higher F_branch_ statistics values. Grey squares are meaningless or redundant comparisons, and white squares are non‐significant comparisons (assuming a significance threshold of 0.05).

## 4 Discussion

### 4.1 Hybridisation in South Atlantic Sea Turtles

With our extensive combined data, we support earlier findings that hybrids are frequent among adult populations of sea turtles in the South Atlantic, specifically crossings involving *Ca. caretta*, *E. imbricata* and *L. olivacea* , species that diverged between 13.6 and 62.8 Mya (Arantes et al. 2025), showing that indeed hybridisation seems to be possible despite long divergence times in animals. We also found that *Ca. caretta* is split into two distinct genetic clusters, the second one (blue samples in Figure 3) appearing in some of the samples that come from the ERG area, an oceanic site known to be a feeding aggregation for multiple populations (nesting sites) of sea turtles (Arantes, Vilaça, et al. 2020; Marcovaldi et al. 2006; Sales et al. 2008).

Additionally, our results suggest that hybridisation is mostly restricted to the first few generations (Figures 2, 3, 4). Thus, further hybrid mating rounds may be rarely successful. Backcrosses with the parental species do take place but appear to be less viable, as indicated by the observation that all backcrossed individuals are found among hatchlings, many of which were stillborn (Arantes, Ferreira, et al. 2020). This intriguing result suggests a degree of hybrid breakdown in these sea turtles, a phenomenon previously hypothesized (Arantes, Ferreira, et al. 2020; Vilaça et al. 2023), but now supported by a large body of high‐quality, reference‐mapped genomic data. Importantly, we also found evidence of an F2 individual (T250BAABPCC) between *Ca. caretta* x *E. imbricata* F1 hybrids that is consistent with the hypothesis of hybrid male fertility published recently (Morais et al. 2026).

The most pervasive hybridisation happens between *Ca. caretta* and *E. imbricata* , especially in the Atlantic coast of the Brazilian state of Bahia, particularly in Praia do Forte, and the Abrolhos archipelago, where hybrid females are known to nest with overall lower success than pure females (Arantes, Ferreira, et al. 2020). Although those species are not directly sister taxa, they have overlapping distributions over their nesting areas in the Brazilian coast and overlapping mating seasons, which could partially explain the high levels of hybridisation found in some areas, e.g., as high as 42% of females in the northern coast of Bahia (Lara‐Ruiz et al. 2006). Notably, if this rate of hybridisation had been ongoing for any significant amount of time, with hybrids having a lower fitness, one could expect the rising of strong prezygotic barriers (e.g., behavioural patterns avoiding hybridisation), a process known as reinforcement. Thus, two scenarios are possible given our data: either hybridisation between the South Atlantic sea turtle species is quite recent, or reinforcement did not occur for some reason. Which scenario is most likely is yet to be elucidated.

Based on previously published (Arantes, Vilaça, et al. 2020; Vilaça et al. 2023) mitochondrial data (Table S1) for 28 samples previously identified as hybrids in the same studies, we can see a clear bias in the directionality of hybridisation. In all crossings between *Ca. caretta* and *E. imbricata* (*N* = 25), including one sample previously identified as a triple hybrid between *Ca. caretta, E. imbricata* and *Ch. mydas*, the individual carried mitochondrial haplotypes typical of *Ca. caretta*, whereas in all crossings involving *L. olivacea* (*N* = 3), they carried haplotypes of the latter species. This data suggests a sex bias, at least when it comes to the first scenario, in which the mother of a hybrid is typically a *Ca. caretta*. However, it is not possible to exclude sampling bias as a factor here, so a wider sampling of mitochondrial haplotypes among hybrids is advised to draw more assured conclusions about directionality in hybridisation events in sea turtles.

Within this study, we also provide molecular evidence of backcrosses between *Ca. caretta* and *L. olivacea* . This implies that F1 hybrids are viable and at least partially fertile, although the lack of adult F2 or backcrossed individuals could be an indication that hybridisation beyond the first generation is deleterious in this species combination.

It is important to note, however, that the observed lack of adult backcrosses and further hybrid generations may be reflective of biases in our sampling instead of a clear biological signal of hybrid breakdown. Therefore, we recommend caution when interpreting these results.

### 4.2 Ancient Gene Flow

With demographic modelling we show that the history of speciation in all four species of sea turtles is shaped by some degree of gene flow since their split from a common ancestor. This indicates that, despite current hybridisation appearing to be limited to the first few generations of crossings, gene flow seems to have played an important role in the speciation of sea turtles. Moreover, our parameter estimates for the asymmetric migration model show that gene flow seems to decrease with divergence time, pointing to a possible “temporal limit” for this phenomenon, when reproductive barriers become more effective. Even though both models of symmetric and asymmetric migration seem to fit the data well, the parameter estimates regarding time of split are more in line with phylogenomic data in the asymmetric migration model (Arantes et al. 2025; Vilaça et al. 2021). In addition to that, we show evidence of long‐term introgression between *Ca. caretta* and *E. imbricata*, and *E. imbricata* and *Ch. mydas*, which suggests that in the past these species hybridised and underwent introgression, leaving a distinct footprint in their genomes.

However, it is important to note that the amount of inferred introgression is very low, which could be an indication that barriers to gene flow arose quickly among those species. This also contrasts with the high levels of hybridisation currently observed, particularly in *Ca. caretta* x *E. imbricata* nesting sites along the Brazilian coastline. Interestingly, we do not find evidence of long‐term introgression between the sister species *Ca. caretta* and *L. olivacea* , despite finding F1 hybrids and backcrosses between those two taxa. Together, this points to a scenario where gene flow in sea turtles was historically possible but rare, which again corroborates the hypothesis of hybrid breakdown currently happening among those species.

Importantly, we urge care when interpreting our modelling results. First, the low sample sizes of the *L. olivacea* and *Ch. mydas* populations could be distorting the SFS and thus biasing model inferences. Second, our limited dataset coming from reduced‐representation sequencing limits the power of our models. Lastly, the amount of inferred gene flow was very low in all comparisons, which begs the question of why a model of gene flow was preferred over a simpler model of isolation—a question we do not address explicitly in this study.

### 4.3 Contrasting Scenarios Regarding Genetic Diversity

All four species of sea turtles seem to be experiencing some level of population recovery thanks to continued efforts in conservation (Fuentes et al. 2023; Hays et al. 2025), which is reflected in our effective population size estimates and Tajima's D results. However, range expansions due to population increase may also increase the probability of hybridisation, which in turn could have negative effects on the recovery of those species (Peñalba et al. 2024), especially in the observed scenario where reinforcement does not seem to occur. Therefore, it is key to take interspecific hybridisation into account when planning conservation strategies for sea turtles, particularly in reproduction sites with different species nesting in overlapping periods.

Our genetic diversity analysis points to contrasting scenarios, in which the green turtle shows high genetic diversity compared to the other three species. In fact, diversity in *Ch. mydas* is more than twice as high as in *E. imbricata* (Arantes et al. 2025), the second most diverse species. This corresponds well to previous estimates of heterozygosity (Vilaça et al. 2021). It is important to investigate if this lower genetic diversity is a long‐term characteristic of these species or a sign of more recent genomic erosion that could lead to the loss of adaptive potential in the future (Fuentes et al. 2023; Hays et al. 2025).

### 4.4 Limitations

We must acknowledge a set of limitations that may impact the conclusions drawn from this study. First, here we used reduced‐representation sequencing libraries which, although powerful and cost‐effective, restrict the number of SNPs that can be recovered, even when using a genomic reference‐based strategy as we did. This can affect analyses that are sensitive to allele dropout and potentially distort the SFS. Second, we recognise that our sampling effort is uneven, with both *Ch. mydas* and *L. olivacea* presenting smaller sample sizes; also, there is a clear geographical bias, where the Brazilian states of Bahia and Sergipe are more well represented than the others. Thus, it is possible that our results are skewed towards reflecting the history of hybridisation and gene flow in that area of Brazil and may negatively affect the general applicability of our work. Lastly, even though the slow evolutionary rate and remarkable genomic conservatism of sea turtles present a methodological advantage, this may limit the translation of our results into systems with stronger divergence.

Nevertheless, we believe our study contributes significant knowledge to the field of population genomics of sea turtles and to the study of hybridisation in vertebrates, using a powerful filtering and reference‐based approach.

## 5 Conclusion

Together, our results point to historical and contemporary hybridisation events between different sea turtle lineages, with a potential for reducing the viability of already endangered populations of these species, possibly due to hybrid breakdown. We also show potential evidence of successful crossings between hybrid males and females. Finally, these results present important insights concerning the regional impacts over the conservation and management of South Atlantic populations of sea turtles, where the overlapping distributions of nesting areas and occurrence of interspecific crossings may potentially lead to a local decline and genetic erosion of these populations over time.

## Author Contributions

M.D.O. has performed research, analysed data, and written the first version of the paper. L.S.A. performed the 3RAD sequencing and data pre‐processing. R.J.P. and C.J.M. contributed with new analytical tools, and C.J.M. contributed with the infrastructure for sequencing. F.R.S. designed research, supervised analyses, wrote the first version of the paper and submitted to the journal. All authors discussed results, revised and approved the final version of the manuscript.

## Funding

This work was supported by Conselho Nacional de Desenvolvimento Científico e Tecnológico, 406864/2022‐5. Fundação de Amparo à Pesquisa do Estado de Minas Gerais, APQ‐04006‐24. Coordinação de Aperfeiçoamento de Pessoal de Nível Superior.

## Disclosure

A research study was developed in Brazil with previously collected tissue samples deposited at the Centro de Coleções Taxonômicas (CCT) of UFMG (Brazil), a public biodiversity collection registered at the Brazilian Ministry of Environment according to the Convention of Biodiversity. All the results of the research are shared with the broader scientific community, concerning the conservation of sea turtles. The access to genetic resources has been also registered at the SisGen platform according to the Nagoya Protocol implemented in Brazil (see Material and Methods).

## Ethics Statement

All the research complies with applicable laws on the study of samples from natural populations available in scientific collections. Because all new data were generated from samples derived from previously collected tissues (between 2004 and 2020) in other projects, which are available in a public collection (CCT‐UFMG), there is no requirement of ethics authorization for animal experimentation.

## Conflicts of Interest

The authors declare no conflicts of interest.

## Supporting information


**Table S1:** Sample metadata: Metadata information for all samples used in the analyses, including previous molecular identification (previous_molecular_id) and mtDNA haplotype assignment (mtDNA).
**Table S2:** Mapping depth: Mean and weighted mean mapping depth per sample as calculated by gstacks.
**Table S3:** ADMIXTURE results: Results from the ADMIXTURE analysis for *K* = 5 and sample classification for the triangle plots labelling.
**Table S4:** Triangle plots Cc × Ei: triangular calculated hybrid index, interclass heterozygosity and missing data for all samples of Ca. caretta, E. imbricata and their hybrids.
**Table S5:** Triangle plots Ei × Lo: triangular calculated hybrid index, interclass heterozygosity and missing data for all samples of E. imbricata, L. olivacea and their hybrids.
**Table S6:** Triangle plots Cc × Lo: triangular calculated hybrid index, interclass heterozygosity and missing data for all samples of Ca. caretta, L. olivacea and their hybrids.
**Table S7:** KING‐robust kinship results: Results from the plink‐calculated KING‐robust kinship values for all pairs of samples.
**Table S8:** Divergence statistics: FST and DXY values for all pairs of species and between the two populations of Ca. caretta.
**Table S9:** dadi model results: Results for all tested dadi models.
**Table S10:** Model parameter estimates: Real estimates of all model parameters for the best models of symmetric migration and asymmetric migration.

**Figure S1:** Distribution of fragment insert size for inward oriented read pairs by sequencing method (3RAD, ddRAD).
**Figure S2:** Overlap of unique mapped positions with > = 5 reads mapped per sequencing method.
**Figure S3:** Weighted mean depth of mapping per sample for each reference genome (primary assemblies only) as reported by *gstacks*.
**Figure S4:** Cross‐validation (CV) error per value of *K* for the best run of each *K* (selected as the lowest CV error among runs). Dashed line shows the value of *K* with the lowest CV error.
**Figure S5:** PCA plot showing PC3 and PC4 for all samples of *Ca. caretta*, *E. imbricata* and *L. olivacea* , confirmed hybrids and non‐identified samples, excluding *Ch. mydas*.
**Figure S6:** Model selection for the demographic analyses. Filled circles represent models with significantly higher likelihoods according to a GIM‐penalised LRT. Cc: *Caretta caretta* ; Cm: *Chelonia mydas* ; Ei: *Eretmochelys imbricata* ; Lo: *Lepidochelys olivacea* .
**Figure S7:** Parameter estimates with confidence intervals for the best fit models of asymmetric migration and symmetric migration. Points are model estimates and bars are the 95% confidence intervals. *m*: migration rate; *nref*: reference population size; *nu1* and *nu2*: effective population sizes of population 1 and 2, respectively; *T*: time since split from the common ancestor.

## Data Availability

Pre‐processed sequencing reads for the 3RAD samples are available for download at DataDryad (https://doi.org/10.5061/dryad.ghx3ffc4s). Code for all analyses is available on GitHub (https://github.com/mdoliv/turtles) and archived on Zenodo (https://doi.org/10.5281/zenodo.23394757).
