# Supplementary material for: Interspecific Gene Flow and Potential Hybrid Breakdown in South Atlantic Sea Turtles
Source: Mol Ecol. 2026 Oct 24;35(20):e70607. doi: 10.1111/mec.70607 (PMC13695011; doi:10.1111/mec.70607)
Supplement: Supplementary file 1 [file MEC-35-e70607-s001.docx]

**Supplemental Information for:**

**Interspecific gene flow and hybrid breakdown in South Atlantic sea turtles**

Oliveira, M. D.; Pereira, R. J.; Arantes, L. S.; Mazzoni, C. J.; Santos, F. R.

**Table of Contents:**

| **Supplementary Figure S1** | Page 2 |
| --- | --- |
| **Supplementary Figure S2** | Page 3 |
| **Supplementary Figure S3** | Page 4 |
| **Supplementary Figure S4** | Page 5 |
| **Supplementary Figure S5** | Page 6 |
| **Supplementary Figure S6** | Page 7 |
| **Supplementary Figure S7** | Page 8 |
| **Table Legends** | Page 9 |

**Supplementary Figure S1:** Distribution of fragment insert size for inward oriented read pairs by sequencing method (3RAD, ddRAD).

**Supplementary Figure S2:** Overlap of unique mapped positions with >= 5 reads mapped per sequencing method.

**Supplementary Figure S3:** Weighted mean depth of mapping per sample for each reference genome (primary assemblies only) as reported by *gstacks*.

**Supplementary Figure S4:** Cross-validation (CV) error per value of *K* for the best run of each *K* (selected as the lowest CV error among runs). Dashed line shows the value of *K* with the lowest CV error.

**Supplementary Figure S5:** PCA plot showing PC3 and PC4 for all samples of *Ca. caretta*, *E. imbricata* and *L. olivacea*, confirmed hybrids and non-identified samples, excluding *Ch. mydas*.

**Supplementary Figure S6:** Model selection for the demographic analyses. Filled circles represent models with significantly higher likelihoods according to a GIM-penalized LRT. Cc: *Caretta caretta*; Cm: *Chelonia mydas*; Ei: *Eretmochelys imbricata*; Lo: *Lepidochelys olivacea*.

**Supplementary Figure S7:** Parameter estimates with confidence intervals for the best fit models of asymmetric migration and symmetric migration. Points are model estimates and bars are the 95% confidence intervals. *m*: migration rate; *nref*: reference population size; *nu1* and *nu2*: effective population sizes of population 1 and 2, respectively; *T*: time since split from the common ancestor.

**TABLE LEGENDS (Supporting Data S1)**

**Table S1 – Sample metadata**: Metadata information for all samples used in the analyses, including previous molecular identification (previous_molecular_id) and mtDNA haplotype assignment (mtDNA).

**Table S2 – Mapping depth:** Mean and weighted mean mapping depth per sample as calculated by *gstacks*.

**Table S3 – ADMIXTURE results:** Results from the ADMIXTURE analysis for K = 5 and sample classification for the triangle plots labelling.

**Table S4 – Triangle plots Cc x Ei:** *triangular* calculated hybrid index, interclass heterozygosity and missing data for all samples of *Ca. caretta*, *E. imbricata* and their hybrids.

**Table S5 – Triangle plots Ei x Lo:** *triangular* calculated hybrid index, interclass heterozygosity and missing data for all samples of *E. imbricata*, *L. olivacea* and their hybrids.

**Table S6 – Triangle plots Cc x Lo:** *triangular* calculated hybrid index, interclass heterozygosity and missing data for all samples of *Ca. caretta*, *L. olivacea* and their hybrids.

**Table S7 – KING-robust kinship results:** Results from the *plink*-calculated KING-robust kinship values for all pairs of samples.

**Table S8 – Divergence statistics:** F_ST_ and D_XY_ values for all pairs of species and between the two populations of *Ca. caretta*.

**Table S9 – *dadi* model results:** Results for all tested *dadi* models.

**Table S10 – Model parameter estimates:** Real estimates of all model parameters for the best models of symmetric migration and asymmetric migration.
